# Changes in language use mediate expressive writing's benefits on health-related quality of life following myocardial infarction

**DOI:** 10.1080/21642850.2014.971801

**Published:** 2014-10-29

**Authors:** David Hevey, Eva Wilczkiewicz

**Affiliations:** ^a^ Research Centre for Psychological Health, School of Psychology, Trinity College Dublin, Dublin, Ireland; ^b^ Department of Cardiology, Beaumont Hospital, Dublin, Ireland

**Keywords:** expressive writing, myocardial Infarction, health-related quality of life, emotional expression, adjustment

## Abstract

The present study assessed linguistic mediators on the effects of expressive writing on health-related quality of life (HRQOL), depression and anxiety following myocardial infarction (MI). One hundred and twenty-one cardiac patients were randomised (expressive writing = 61; control = 60), 98 (expressive writing = 47; control = 51) provided pre- and post-data, with 89 (expressive writing = 43; control = 46) completing the three-month follow-up. The expressive writing group wrote (20 mins/day for three consecutive days) about their thoughts and feelings regarding their MI, and the control group wrote (20 mins/day for three consecutive days) about daily events that occurred during the year prior to the MI. The outcome measures of depression, anxiety and HRQOL were completed pre-randomisation, post-intervention and three months post-intervention; the mediating variables assessed were changes in (a) positive emotion words, (b) negative emotion words and (c) cognitive-processing words. Three months post-intervention, the expressive writing group had significantly higher HRQOL. The positive effects of expressive writing were significantly associated with increases in both positive emotion words and cognitive-processing words across the three days of expressive writing. Expressive writing is a beneficial intervention that may enhance HRQOL among cardiac patients.

## Introduction

Cardiovascular disease (CVD) is the number one cause of death globally (WHO, 2011). Advances in the management of acute cardiac events such as myocardial infarction (MI) have resulted in increasing numbers of cardiac patients requiring continuing care; enhancing secondary prevention and improving health-related quality of life (HRQOL) are core goals for such care (WHO, 1993). HRQOL is a subjective measure of overall well-being, and although numerous definitions and measures of HRQOL exist, current assessment approaches tend to focus on social, physical and psychological functioning (Swenson & Clinch, 2000). HRQOL assessment contributes to the clinical management of cardiac patients by providing an additional and complementary measure to traditional standard biomedical outcomes such as blood pressure (Wilson & Cleary, 1995). A number of HRQOL scales have been developed specifically for use with cardiac patients and assess the three core domains of social, physical and psychological functioning, for example, the MacNew Heart Disease health-related quality of life instrument (Valenti, Lim, Heller, & Knapp, 1996).

CVD patients report impaired HRQOL compared to members of the general population (De smedt et al., 2013; Xie et al., 2008), and research has shown a significant association between HRQOL and long-term outcomes. For example, declines in HRQOL after coronary artery bypass grafting (CABG) predict later adverse cardiovascular events (Gunn, Lautamäki, Hirvonen, & Kuttila, 2014). Furthermore, even after standard risk factors (i.e. demographic factors such as age, clinical factors such as medical comorbidities, cardiac risk factors such as hypertension, and CVD severity) have been controlled for, poor HRQOL predicted morbidity and mortality in CVD patients (Spertus, Jones, McDonell, Fan, & Fihn, 2002). Affective disorders (depression and anxiety) are common within cardiac populations, and are significantly associated with impaired HRQOL (Rozanski, Blumenthal, Davidson, Saab, & Kubzansky, 2005).

In the CVD research literature depression typically refers to unipolar clinical depression, as diagnosed using established psychiatric criteria through a clinical assessment, and depressive mood, as measured by a standardised self-report scale (Goldston & Baillie, 2008). High scores on depressive mood scales are strongly correlated with the presence of unipolar clinical depression (Williams, Pignone, Ramirez, & Perez Stellato, 2002). Compared to the general population, depression is common in cardiac patients: extensive research evidence indicates that prevalence rates of depression in CVD patients are 20% or higher (Krantz & McConey, 2002). Depression is not only an independent risk factor in the etiology of CVD, but it also impacts negatively on the prognosis for patients, increasing both the risk of further cardiac events and mortality; the risk remains elevated after controlling for factors such as age, previous MI, cardiovascular health, medical comboridities such as diabetes and hypertension (Van Melle et al., 2004). Of note, a recent systematic review reported a significant association between depression and impaired physical HRQOL in cardiac patients (Dickens, Cherrington, & McGowan, 2012).

Similar to depression, the anxiety disorders are over-represented in cardiac patients compared to community populations (Harter, Conway, & Merikangas, 2003). Anxiety disorders comprise a number of clinically defined disorders (e.g. panic disorder, social phobia, generalised anxiety disorder, post-traumatic stress disorder); however, many of the anxiety scales used in research measure a broad range of features associated with anxiety in general rather than specific disorders (Kubzansky, Kawachi, Weiss, & Sparrow, 1998). In a recent meta-analysis, anxiety was identified as an independent risk factor for the incidence of CVD and cardiac mortality in initially healthy individuals, after adjusting for a variety of demographic variables (e.g. age), biological risk factors (e.g. body mass index, blood pressure, cholesterol) and health behaviours (e.g. smoking) (Roest, Martens, De Jonge, & Denollet, 2010). For example, elevated anxiety symptoms were recently associated with a two-fold increased risk of mortality in CVD patients (Watkins et al., 2013); of note, the increased mortality risk was strongest in CVD patients with comorbid anxiety and depression, in whom the risk of mortality was three-fold higher than in patients with neither anxiety nor depression. A recent review concluded that anxiety predicts poor HRQOL in newly diagnosed cardiac patients (Pragodpol & Ryan, 2013).

Research evidence suggests that HRQOL among cardiac patients is impaired by depression and anxiety (Williams, 2008); consequently psychosocial interventions may be required to facilitate patient adjustment post-MI. The present study examines if expressive writing is an effective intervention for this population.

### Expressive writing

The use of writing in a therapeutic manner derives from psychotherapeutic traditions characterised by interpersonal disclosure, which includes identifying, labelling and disclosing emotional experiences (Smyth & Helm, 2003). In expressive writing research, participants in the experimental group typically write about traumatic experiences or stressful events, whereas participants in a control group write about trivial subjects (Pennebaker & Chung, 2007). Individuals in both groups are instructed to write without regard to spelling, style or grammar, and are informed that their written narratives remain conﬁdential. Each writing session takes approximately 15–30 min each day for several consecutive days. Although initial studies allowed participants to change the topics for each writing session, many studies have required participants to write about a specific event across all sessions to facilitate cognitive processing of the event (Frattaroli, 2006). Such processing and expression of previously suppressed emotions often results in decreased physiological arousal, and subsequent mental and physical health benefits (Pennebaker, 1997).

Early research examined the effect of expressive writing in student samples, and reported improvements in mood and healthcare service use (Pennebaker & Beall, 1986). A number of studies have examined the generalisability of effects to various clinical populations. For example, patients with chronic asthma or rheumatoid arthritis (RA) were randomly assigned to write about either stressful life events or a neutral topic (time management) (Smyth, Stone, Hurewitz, & Kaell, 1999). Four months after treatment, asthma patients in the experimental group showed improvements in lung function (increased mean percentage of predicted forced expiratory volume in 1 second [FEV_1_]), whereas control group patients showed no change. RA patients in the experimental group showed improvements in overall disease activity, whereas control group patients did not change. Although some studies reported benefits for other clinical populations including breast cancer (Stanton et al., 2002), HIV infection (Petrie, Fontanilla, Thomas, Booth, & Pennebaker, 2004) and fibromyalgia (Broderick, Junghaenel, & Schwartz, 2005), other clinical studies have failed to report benefits for patients with the same condition (e.g. breast cancer: Walker, Nail, & Croyle, 1999) or different conditions (e.g. asthma; Harris, Thoresen, Keith, & John, 2005). Given such discrepancies in findings, Frisina, Borod, and Lepore (2004) performed a meta-analysis on the effects of structured writing in medical populations. For patients with medical/physical disorders, an overall signiﬁcant beneﬁt was observed (*d* = 0.21). A later meta-analysis of expressive writing randomised controlled trials (RCTs) by Frattaroli (2006) reported an overall effect size (ES) *d* of 0.15; although these ESs may seem small, it is important to note that the fact that an effect is present at all is impressive given the simplicity of the intervention in terms of administration, the minimal cost (pens, paper and envelopes) and length of time devoted to writing (i.e. one hour total), and the absence of formal supervised guidance from a healthcare professional (Frattaroli, 2006).

### Expressive writing and CVD

McGuire, Greenberg, and Gevirtz (2005) noted that one month after writing, participants with elevated blood pressure who completed expressive writing exhibited lower systolic and diastolic blood pressure (DBP) than before writing. Four months after writing, DBP remained lower than baseline levels. Recently, in an RCT comparing the effects of expressive writing (writing about innermost thoughts and feelings about an MI) with a writing control (writing about an emotionally bland, trivial topic), expressive writing was associated with better physical and mental health among patients after their first MI (Willmott, Harris, Gellaitry, Cooper, & Horne, 2011). They also reported that expressive writing had clinically beneficial effects, for example, fewer General Practitioner and hospital visits, less prescribed medication and fewer reported cardiac symptoms.

### Mechanisms

Determining the mechanisms by which expressive writing benefits individuals is important as such information can be used to enhance the active therapeutic processes to improve the likelihood of beneficial outcomes. Although a number of different theories have been proposed to explain the beneficial effects of writing (see Pennebaker & Chung, 2007), the present study examines the emotional expression and cognitive-processing aspects of written disclosure.

The initial expressive writing studies (Pennebaker & Beall, 1986) were predicated on the idea that writing allowed participants to express previously inhibited thoughts and emotions in response to a trauma/stressful event; by lifting inhibitions against expression and by allowing previously avoided material to be processed and organised, such expression would have psychological and physical health benefits. Notably, the expression of emotions associated with the events was seen as critical: describing only the facts of a trauma was not beneficial (Pennebaker & Beall, 1986). However, the mere emotional expression of a trauma is not sufficient (Krantz & Pennebaker, 2007), and translating experiences into language appears to be essential for the writing to be effective. Cognitive-processing theory emphasises the need to make sense of, organise and integrate thoughts and feelings about an event to accrue benefit (Pennebaker, 1993).

Cognitive restructuring during expressive writing is critical and Tausczik and Pennebaker (2010) argue that words people use provide important psychological cues to their thought processes and emotional states; consequently the role of language in the processing of traumatic/stressful events has become a core focus. Pennebaker and Chung (2011) argue that translating an event into language allows the person to assign meaning, coherence and structure to the event, which facilitates assimilation of the event: such assimilation may enhance coping responses, and subsequently result in better adjustment post-event. Greater use of causal and insight words facilitates the building of a narrative framework over the course of writing, which may result in a reappraisal of the experience in a less threatening manner (Pennebaker & Chung, 2011). This process may consequently result in a reduction of negative emotions, as indexed by less use of negative emotion words, arising when thinking and writing about the experience. Pennebaker and Chung (2007) suggested that using insight and causal words in conjunction with positive emotion words is a language pattern that reflects a positive reappraisal of the experience, which may facilitate cognitive broadening (Fredrickson, 2001) to produce expressive writing's benefits.

Other researchers have examined expressive writing from an emotional exposure–habituation model (Sloan & Marx, 2004), which argues that it is habituation to negative affects and intrusive thoughts about a traumatic event that produce the positive effects. Habituation to emotional stimuli has been extensively examined using behavioural therapies (Meadows & Foa, 1999); such research illustrates how a classically conditioned link between an event and the individual's reaction to it can be extinguished during repeated exposure. However this process also requires cognitive processing as people may change in their understanding and representations of the event (Pennebaker & Chung, 2007). Expressive writing focuses attention on thoughts and feelings surrounding stressful events, and as participants describe the stressful experience (an aversive stimulus) negative affect is also evoked through the memory of the experience (Lepore, Greenberg, Bruno, & Smyth, 2002). However, by repeatedly exposing the individual to the aversive stimulus, expressive written disclosure may facilitate habituation to both stimulus and the negative affective response, consequently extinguishing negative emotional associations, which produces beneficial outcomes (Lepore, 1997). Cognitive processing of the event would result in a reappraisal of the event. Such habituation of negative affect during the course of expressive writing sessions has been reported in the literature (Sloan & Marx, 2004) and decreases in negative emotion word use would reflect such a process.

The mechanisms underlying expressive writing are still under investigation. Analysing the patterns of word use might reveal the cognitive and emotional processes that occur during expressive writing; in line with Shim, Cappella, and Han (2011), it is plausible that changes in word use might reflect different theoretical processes. Increases in causal/insight and positive emotions words may bring benefits through cognitive adaptation, whereas changes in negative emotion word use may be beneficial in terms of an emotional exposure–habituation process.

### Linguistic inquiry and word count

Pennebaker, Francis, and Booth (2001) developed a computer programme called Linguistic Inquiry and Word Count (LIWC), which quantiﬁes the language statistics (e.g. word count, sentence length) and linguistic dimensions (e.g. pronoun use) of written text. LIWC goes through each line of written text word by word and each word is categorised by comparing it to all the words present in LIWC's extensive dictionary file, which has evolved with each version of LIWC. High levels of correlations have been reported between LIWC's coding of words and ratings made by external judges (Tausczik & Pennebaker, 2010). LIWC can code words into “content words” (e.g. nouns, regular verbs, adjectives, adverbs) and “style words” (e.g. pronouns, prepositions, articles). Of particular relevance, LIWC can also provide information about psychological processes by measuring different categories of relevant words in a text. It counts the frequency of positive emotion (e.g. “happy” “love”, “nice”) and negative emotion (e.g. “worthless”, “hurt”, “ugly”) words; in addition it measures words that signify cognitive processing (e.g. causal words such as “because” or “cause”, and insight words such as “think” or “consider”).

Pennebaker (1993) argues that the effect of writing allows for the development of reappraisal, understanding and the creation of a coherent narrative of the link between cognition and emotion. In a study of the impact of journaling about stressful events (writing at least twice a week for at least 10 minutes over a month), Ullrich and Lutgendorf (2002) showed a mediating effect of cognitive processing: those who used more cognitive-processing words benefited most. Other studies have similarly reported that expressive writing's effects are strongest for those essays with an increased use of these cognitive-processing words over time (Pennebaker, 1993; Petrie, Booth, & Pennebaker, 1998; Rivkin, Gustafson, Weingarten, & Chin, 2006). Tausczik and Pennebaker (2010) propose this pattern of change in cognitive-processing words indicates the development of a narrative in which participants begin actively to process and reappraise events. The results from studies exploring the use of emotion words have been more mixed, with health improvements being associated with greater use of positive emotion words in some studies (Danner, Snowdon, & Friesen, 2001) but negative emotion words in others (Pennebaker, 1993). In general, expressive writing may be most beneficial if the experience described evokes emotional responses while also facilitating cognitive processing.

### Aims and objectives

The present study assessed the effects of expressive writing on HRQOL following MI. In addition, potential linguistic mediators of the effects were examined. It was hypothesised that

- expressive writing would be associated with enhanced HRQOL and decreased depression and anxiety
- expressive writing's effects on HRQOL, depression and anxiety would be mediated by increases in both positive and cognitive-processing word use, and decreases in negative emotion word use during the writing intervention.

## Method

### Sample

Consecutive patients with a confirmed MI who received treatment at a large teaching hospital were invited to participate. Exclusion criteria included an inability to understand English or write for 20 minutes, and a history or current evidence of psychiatric illness (determined by examination of medical charts). The sample, with a mean age of 61.7 years (SD = 8.7), was predominantly male (*n* = 68; 76%). Approximately half (*n* = 42; 47%) of the participants completed only primary level of education and a further third (*n* = 29; 33%) completed secondary level. The majority lived with others (*n* = 82; 92%) and over one-third (*n* = 33; 37%) were in full-time employment. Forty-three per cent had a previous cardiac event (CABG, *n* = 42, 47%; Percutaneous Coronary Intervention *n* = 25, 28%; MI, *n* = 15, 17%; other *n* = 7, 8%). In terms of co-morbidities, one-third (*n* = 30; 34%) had hypertension and 8% (*n* = 7) had diabetes.

### Procedure

Following receipt of IRB approval from the research institution and hospital, the lead researcher approached eligible patients in the week following their admission to hospital. An information letter describing the study was provided and those who agreed to participate signed the consent form. Demographic details (e.g. gender, age) and medical history, including history of psychiatric illness, were recorded. At discharge from hospital participants were randomly assigned to either the intervention or control group, and subsequently received an envelope containing the instructions for writing sessions. All writing sessions were for 20 minutes per day for three consecutive days.

A standard procedure for expressive writing was used (Pennebaker & Chung, 2007) wherein participants were asked to write at home in a quiet room preferably on the paper supplied for 20 min and then place their sheet in the supplied envelope. In line with previous studies those in the intervention condition were asked to write over the three days about their deepest thoughts and feelings in relation to having had a heart attack. Participants were encouraged to “really let go and explore your very deepest feelings and thoughts”. They were told that they might relate the heart attack to “your childhood, relationships with others, including parents, lovers, friends or relatives; to your past, your present or your future; or to who you have been, who you would like to be or who you are now”. They were informed that in the act of writing they may provoke a strong emotional response and that such a response was normal. The control group described daily activities in the year prior to their heart attack. They were instructed to describe their activities, and to avoid writing about their thoughts and feelings about the activities. They were encouraged to “Please write as objectively as possible”.

### Measures

The *Mac New Health-Related Quality of Life* (Valenti et al., 1996) scale is a 27-item measure of physical, emotional and social QOL. The items were generated through interviews with physicians, nurses, allied health professionals, patients with MI and by reviewing the literature. All items are rated on seven-point Likert scale and in addition to separate physical, emotional and social QOL subscales, an overall index of QOL is provided. Higher scores indicate higher levels of HRQOL. It has been extensively used in cardiac populations, and a review concluded that there was evidence supporting the scale's factorial validity, construct validity, reliability and sensitivity to change over time (Höfer, Lim, Guyatt, & Oldridge, 2004). Based on data from over 1000 cardiac patients a change of 0.5 units reflects a minimal clinically important difference (Dixon, Lim, & Oldridge, 2002). In the present sample a Cronbach's alpha of 0.93 was found for the overall HRQOL index.

The *Hospital Anxiety and Depression Scale* (HADS, Zigmond & Snaith, 1983) is a widely used 14-item instrument developed to detect states of anxiety (7 items) and depression (7 items) in medical outpatients. It was developed to identify clinical caseness (possible and probable) of anxiety disorders and depression among patients in non-psychiatric hospital clinics. Of note, in order to avoid potential confounding by somatic illness factors, the HADS focuses on psychological and cognitive symptoms, rather than symptoms of anxiety or depression relating also to a physical disorder (e.g. dizziness, headaches, insomnia, anergia and fatigue). A cut-off score of 8 on the HADS-A subscale (range 0–21) and 8 on the HADS-D subscale (range 0–21) has been found to provide the optimal balance between sensitivity and specificity for identifying possible clinical cases (Bjelland, Dahl, Haug, & Neckelmann, 2002). The psychometric properties (reliability, factorial validity, discriminant validity, sensitivity and specificity) and the clinical utility of the HADS in cardiac populations have been established (Bjelland et al., 2002). In the present sample the Cronbach's alpha for the anxiety scale was 0.84 and for the depression scale was 0.81.

### Analysis

The written essays for each day for each participant were typed into individual Microsoft Word files according to the Operator's Manual for LIWC (Pennebaker, Booth, & Francis, 2007). LIWC uses large, independently validated dictionaries to assign the essay words into predefined categories of positive emotion words, negative emotion words and cognitive mechanism words.

Based on the G*Power 3.1.2 (Faul, Erdfelder, Lang, & Buchner, 2007) software programme, a sample size of 60 in each of the two groups assessed at three time-points (pre-intervention, post-intervention and three months post-intervention) provided power of 0.90 to a mixed ANOVA to detect a small-sized effect (*f* = 0.10) as being significant at the 0.05 level.

Data were analysed using SPSS version 21. At baseline between-groups comparisons were made using independent samples' t tests for continuous variables and chi-square tests for categorical data. To examine the effectiveness of the intervention both *intention-to-treat* and *as treated* analytical protocols were used. The *intention-to-treat* analysis was performed using SPSS Mixed Models, with an autoregressive error term (given the repeated measurement design) and restricted maximum likelihood estimation specified to examine the effects of condition (intervention vs. control group), time (scores at each of the three time-points: pre-intervention, post-intervention and three months post-intervention) and the condition by time interaction. The *as treated* analysis was conducted using mixed ANOVA with condition as the between-subjects variable, and time as the within-subjects variable. To examine the mediation effects data were only used from participants in the Expressive Writing condition who adhered to treatment protocols (i.e. completed three written essays) as calculation of the change in language use over the course of the writing required time 1 and time 3 essays to be completed. Correlations examined whether changes in language use (i.e. increase in cognitive-processing/positive emotion words, decrease in negative emotion words) were associated with changes in HRQOL, depression and anxiety. For all analyses statistical significance was set at 0.05 and ESs are reported using Cohen's *d* or *η* ^2^.

## Results

One hundred and seventy-eight eligible participants were approached and invited to participate. Of these, 46 declined the offer to participate, eight had clinical complications and three who had originally agreed to participate asked to be withdrawn; consequently 121 (response rate of 68%) cardiac patients were randomised (Intervention = 61; Control = 60), 98 (Intervention = 47; Control = 51) provided pre- and post-data, with 89 (Intervention = 43; Control = 46) completing the three-month follow-up. There were no significant differences between those who completed time 1 only and those who provided complete data in relation to clinical, demographic and psychosocial variables. Similarly, there were no significant differences between the Intervention and Control groups regarding clinical and demographic variables at time 1.

### Baseline psychosocial status

The sample at time 1 can be characterised in terms of moderate levels of HRQOL (the scale *M* was close to the scale midpoint for both the Expressive Writing (*M* = 4.4 [SD = 0.9]) and Control (*M* = 4.2 [SD = 1.0]) groups). There were low levels of distress for both groups in relation to depression (Expressive Writing *M* = 4.3[SD = 3.1] and Control *M* = 4.2[SD = 2.9]) and anxiety (Expressive Writing *M* = 6.3[SD = 3.3] and Control *M* = 6.3 [SD = 3.1]). Independent samples t-tests revealed no significant differences between the groups in relation to HRQOL, depression or anxiety. Furthermore, there were no differences between the groups regarding the number of patients scoring in the clinical range on the HADS for depression (Expressive Writing *n* = 6, 14%; Control *n* = 5, 11%; *χ* ^2^ (1) = 0.02, *p* > 0.05) or anxiety (Expressive Writing *n* = 8, 18%; Control *n* = 6.14%; *χ* ^2^ (1) = 0.18, *p* > 0.05).

### HRQOL

The *intention to treat* analysis revealed no main effect of group, *F*(1121.16) = 1.9, *p* > 0.05. Both the main effect of time, *F*(1188.10) = 18.7, *p* < 0.001, and the interaction were statistically significant, *F*(2188.10) = 4.1, *p* < 0.001. Of note the *as treated* analysis similarly revealed that the main effect of group was not significant (*F*(1, 87) = 2.1, *p* > 0.05) and that the effect of time was significant (*F*(2138.15) = 21.2, *p* < 0.001, *η* ^2^ = 0.34). Using a Bonferonni correction the overall means at time 1 (*M* = 4.39), time 2 (*M* = 4.95) and time 3 (*M* = 5.56) were all significantly (*p* < 0.01) different to each other, with HRQOL increasing over time. The interaction was statistically significant, *F*(2138.15) = 4.3, *p* < 0.05, *η* ^2^ = 0.05, and *post hoc* analyses revealed that three months post-intervention, the Expressive Writing intervention group had significantly higher total HRQOL than the control group, *t*(87) = 2.04, *p* < 0.05, Cohen's *d* = 0.42. The mean change in HRQOL for the intervention group exceeded the minimal clinical significant change criteria for time 1 to time 2 (*M* change = 0.57) and for time 1 to time 3 (*M* change = 1.15). No other between-group comparison was significant.

### Depression

The *intention to treat* analysis revealed no main effect of group (*F*(1121.16) = 0.83, *p* > 0.05) and no interaction effect (*F*(2188.10) = 0.92, *p* > 0.05). The main effect of time, *F*(2188.10) = 16.54, *p* < 0.001, was statistically significant. The same pattern emerged using the *as treated* analysis. The main effect of group was not significant, *F*(1, 87) = 0.94, *p* > 0.05; however, the effect of time was significant: *F*(2133.58) = 19.64, *p* < 0.001, *η* ^2^ = 0.31. Using a Bonferonni correction the overall mean at time 1 (*M* = 4.2) was significantly (*p* < 0.01) higher than the mean at time 3 (*M* = 3.3). The interaction was not statistically significant, *F*(2138.15) = 0.86, *p* > 0.05.

### Anxiety

The *intention to treat* analysis revealed no main effect of group (*F*(1121.16) = 1.46, *p* > 0.05) and no interaction effect (*F*(2188.10) = 1.01, *p* > 0.05). The main effect of time, *F*(2188.10) = 21.39, *p* < 0.001, was statistically significant. The same pattern emerged using the *as treated* analysis. The main effect of group was not significant, *F*(1, 87) = 1.52, *p* > 0.05; however, the effect of time was significant: *F*(2150.73) = 23.88, *p* < 0.001, *η* ^2^ = 0.34 . Using a Bonferonni correction the overall mean at time 1 (*M* = 6.2) was significantly (*p* < 0.01) higher than the mean at time 3 (*M* = 4.9). The interaction was not statistically significant, *F*(2150.73) = 1.17, *p* > 0.05.

### Mediation analyses

As there were no significant effects in relation to depression or anxiety, mediation analyses only examined the relationship between changes in word use by the expressive writing group and HRQOL. Among those in the expressive writing condition, greater improvement in HRQOL was associated with greater increases from the first to the third writing session in use of positive words (*r* (39) =  0.37, *p* < 0.05) and cognitive-processing words (*r* (39) =  0.32, *p* < 0.05).

## Discussion

Using both *intention to treat* and *as treated* analyses expressive writing was associated with improvements in HRQOL among a sample of post-MI patients. The pre- to post-intervention mean improvement in HRQOL for the expressive writing group met the currently accepted criterion for indicating clinically meaningful change (i.e. mean change of 0.50 units); indeed the improvement from time 1 to time 3 (mean change = 1.15) was over double this criterion (Dixon et al., 2002). However, the intervention had no significant effects for either anxiety or depression; this may be due to the relatively low levels of distress present in the sample. For example the depression rate in the current study was 12%, whereas a higher average prevalence rate of 16% was reported in a systematic review of studies using the HADS to assess depression among cardiac patients (Thombs et al., 2006). Similarly, the rate of anxiety in the current sample (16%) is lower than that reported in previous studies with cardiac patients; for example, Frasure-Smith and Lesperance (2008) reported that 41% of a sample of over 800 cardiac patients scored above the cut-off value for the HADS anxiety scale. In addition, the significant main effects of time suggest that the changes in distress may reflect the natural course of symptom resolution over time.

The HRQOL results are consistent with those of Willmott et al. (2011), who reported benefits of MI-focused expressive writing 5 months post-intervention, relative to the control group in relation to having significantly fewer recorded medical appointments, lower levels of prescribed medicines, greater attendance at cardiac rehabilitation sessions, fewer cardiac-related symptoms and lower diastolic blood pressure. Thus the present study's findings add to the nascent body of research on the effects of expressive writing for CVD patients; of note the ongoing WRITTEN-HEART (Manzoni, Castelnuovo, & Molinari, 2011) study is currently investigating the feasibility, safety, clinical efficacy and cost effectiveness of expressive writing in 92 coronary heart disease (CHD) patients referred to CR. It is an RCT comparing the effects of four conditions (expressive writing about having heart disease vs. expressive writing about a traumatic/negative life event *vs*. neutral writing about the facts regarding CHD and its treatment *vs*. a non-writing condition) on HRQOL, depression, anxiety and post-traumatic growth at 3, 6 and 12 months post-discharge from CR. If additional positive effects are reported, it may be useful to consider implementing expressive writing in routine clinical context for cardiac patients; such an approach could be based on the model of weekly expressive writing interventions delivered in a busy oncology clinic (Morgan, Graves, Poggi, & Cheson, 2008).

Mediation analyses revealed that expressive writing's beneficial effect on HRQOL was mediated by increases from the first writing session to the third writing session in positive emotion words and cognitive-processing words. Previous research has also reported that the more that people use positive emotion words, the more their health improves (Pennebaker & Chung, 2011) and that the more that people use causal and insight words, the more they benefit from expressive writing (Pennebaker, Mayne, & Francis, 1997; Ullrich & Lutgendorf, 2002). The MI-focused writing may help to structure traumatic memories of the MI, producing a coherent narrative that is necessary for expressive writing to be beneficial (Smyth, True, & Souto, 2001). The changes in cognitive-processing words and positive words are in line with predictions from the cognitive restructuring accounts of expressive writing: the linguistic changes may reflect the individual's development of a structured and cohesive narrative, which promotes insight and assimilation of traumatic memories (Pennebaker, 1990). The failure to find an effect of negative emotion word use suggests that habituation to the negative affect associated with the MI may not have occurred; other studies have similarly failed to find mediating effects of changes in negative emotion word use (Slatcher & Pennebaker, 2006). However, as noted previously the present sample was characterised by a low level of distress/negative affect and this may account for the lack of change in negative emotion word use. In general, it must be acknowledged that to date, there is still widespread uncertainty over the mechanisms for expressive writing's benefits, with little consensus in the empirical literature.

Expressive writing's effects are potentially moderated by a number of variables (Frattaroli, 2006). For example expressive writing was beneficial for those high in Alexithymia (O'Connor & Ashley, 2008) and Type D (Hevey, Wilczkiewicz, & Horgan, 2012); other potential individual difference variables could be investigated in the context of cardiac patients. Although typical expressive writing instructions ask participants to focus on negative events, improved outcome has also been associated with methods that ask participants to focus on positive aspects of such events. For example, Stanton and colleagues (2002) found that asking patients with breast cancer to explore through writing the potential beneﬁt associated with their experiences led to reductions in both physical symptoms and symptom-related medical visits. In addition, Cameron and Nicholls (1998) demonstrated that focusing writing around future coping strategies effectively reduced healthcentre visits and improved reported affect in college students. Additional research examining the effectiveness of different variants of the expressive writing task is required to inform optimal task focus. Based on the present findings research could examine the effectiveness of a method that explicitly asked participants to increase their positive and cognitive-processing word use. In addition, given recent research supporting the benefits of Cognitive Behavioural Therapy (CBT) among cardiac patients (Doering et al., 2013; Gulliksson et al., 2011; Hwang et al., 2014) and Dickens et al.’s (2013) conclusion recommending CBT from a systematic review of psychological interventions for depression with cardiac patients, it may be productive to examine the extent to which CBT can stimulate changes in cognitions and emotions that produce changes in the use of positive and cognitive-processing words.

### Limitations

The applicability of findings is limited by the relatively small sample size and attrition rate, and caution regarding generalisability of the present results is warranted until further replication studies are conducted. In addition, the relatively low levels of psychological distress limit the generalisability of the findings to more distressed CVD populations. The uptake rate of the study (68%) compares favourably to those reported in the literature (Willmott et al., 2011); however it suggests that the intervention may not be acceptable to one-third of the eligible population. The high attrition rate is also a limitation: although no significant differences emerged between those who remained in the study and those who dropped out, these groups may differ on variables not assessed. For both groups, approximately one-third of those assigned to the group dropped out. Research examining reasons for attrition would be beneficial. Daily reminders were not sent to participants to complete their writing assignments; the use of such prompts in future research may enhance adherence to the intervention protocol.

The paper presents both *intention to treat* and *as treated* analyses in the Results as the focus of the study was on examining the effects of completing the writing tasks on HRQOL and the mediating effects of changes in language use. As intention to treat may provide a test of treatment allocation rather than the actual treatment *per se*, we conducted both approaches (Petkova & Teresi, 2002). Medical records and self-reports assessed current and previous psychiatric illness; it would be beneficial to screen for psychiatric illness using standardised diagnostic interviews in future studies. The follow-up period is quite short and it remains to be determined if HRQOL benefits are maintained long term. A larger sample size with the inclusion of clinical outcomes (e.g. healthcare utilisation) and physiological variables would determine the intervention's clinical significance. Such research could also examine the cost-effectiveness of expressive writing. Indeed, Mc Guire, Greenberg, and Gevirtz (2005) argued that a low-cost and easily administered psychological intervention such as expressive writing, if shown to be effective, has the potential for widespread clinical use. However, to-date such analyses have not been reported among cardiac patients.

## Conclusion

The present positive findings support the growing literature of expressive writing studies with clinical populations (Frisina et al., 2004). Expressive writing is a potentially beneficial intervention that may facilitate adjustment post-MI.
